# Physicians' Perceptions and Adherence to Guidelines for the Management of Hypertension: A National, Multicentre, Prospective Study

**DOI:** 10.1155/2012/503821

**Published:** 2012-11-28

**Authors:** Mamas Theodorou, Panagiotis Stafylas, Georgia Kourlaba, Daphne Kaitelidou, Nikos Maniadakis, Vasilios Papademetriou

**Affiliations:** ^1^Faculty of Economic Sciences and Management, Open University of Cyprus, 1304 Nicosia, Cyprus; ^2^Hypertension Unit, 1st Department of Internal Medicine, AHEPA University Hospital, 54636 Thessaloniki, Greece; ^3^Department of Health Services Organization and Management, National School of Public Health, 196 Alexandras Avenue, 11522 Athens, Greece; ^4^Centre for Health Services Management and Evaluation, Department of Nursing, University of Athens, 11527 Athens, Greece; ^5^Veterans Affairs Medical Center, Georgetown University, Washington, DC 20422, USA

## Abstract

*Background*. The aim of the current study was to investigate physicians' perceptions and adherence to the European guidelines for the management of hypertension. *Methods*. This is a national, multicentre, prospective, observational study, conducted between November 2007 and June 2008, in Cyprus. Consecutive hypertensive patients have been recruited by a random sample of physicians. The physicians' recommendations for every single patient have been recorded and compared with the 2007 ESH/ESC guidelines. *Results*. Of the total of 654 patients, 477 (72.9%) were correctly advised by their physician to receive antihypertensive treatment to control their blood pressure, while 396 (60.5%) correctly got advices to adopt only lifestyle changes. The overall adherence of physicians to the European guidelines (overall agreement rate) was 70.4% (*k* = 0.258, *P* < 0.001). Of the total of 68 physicians, 65 (95.6%) reported that they were aware of some guidelines. There was no statistically significant effect of specific physicians' characteristics on the overall adherence to guidelines, but there was in the percentage of patients achieving medication guidelines. *Conclusions*. The study demonstrated that although Cypriot physicians declared that they were aware of the clinical guidelines for the management of hypertension, more than one-fourth of high risk hypertensive patients remained untreated and 40% of low risk patients received inappropriate medication.

## 1. Introduction

Hypertension is a highly prevalent condition affecting more than one-third of the world population [1, 2]. Moreover, it is a major modifiable risk factor for cardiovascular disease (CVD) and a leading cause of mortality and disability-adjusted life years (DALYs) [3, 4]. Although several studies have revealed that blood pressure lowering strategies substantially reduce the CVD risk [5, 6], other studies have showed that the most of the hypertensive patients remain uncontrolled [1, 7–10]. It is well established that the observed poor control of the disease is largely attributed to the poor patients' adherence to medical advice and recommended medication [11, 12]. However, physicians' awareness and adherence to evidence-based management of hypertension (physician inertia) are equally important aspects of the same problem, but they have not been adequately studied [13].

Guidelines aim to assist health care providers in determining appropriate management of hypertension in order to increase the percentage of patients with controlled blood pressure. Scientific associations, such as European Society of Hypertension (ESH) and others [14–16] have published guidelines for the management of arterial hypertension summarizing the data from large randomized clinical trials and other sources and offering the best available, balanced, and comprehensive recommendations to all health care providers. The cost-effectiveness of these recommendations has been demonstrated in published studies worldwide [17, 18]. However, although no established methodology exists to assess physicians' adherence to guidelines for management of hypertension, a great number of studies have documented a low physicians' compliance to recommendations by following different methodologies [19]. Moreover, studies tend to describe overall level of adherence but the physicians' or patients' characteristics that influence the acceptance and implementation of the guidelines are not as well studied [13].

This is particularly true in Cyprus, where cardiovascular morbidity and mortality are comparable with other European Union countries (EU15) [20]. Therefore, the primary aim of the current study was to investigate the self-reported attitudes of Cypriot physicians' towards the usefulness of guidelines as well as to quantify the level of adherence to the guidelines for the management of hypertension. The secondary aim of this study was to examine the extent to which characteristics of the physicians or the patients affect physicians' adherence to evidence-based medicine.

## 2. Methods

### 2.1. Study Design and Participants

This is a national, multi-centre, prospective, observational study, conducted between November 2007 and June 2008 [21] on the island of Cyprus. The population under study was comprised of patients with hypertension examined by a random sample of physicians located in all Cyprus districts (Nicosia, Limassol, Larnaca, and Paphos). The primary target was that at least 10% of all the Cypriot physicians involved in the management of hypertension would participate in the study. Based on the collaboration of the Ministry of Health, of the Cyprus Society of Hypertension (CSH), and the Cyprus Society of Cardiology (CSC) and a participation fee, it was assumed that a high participation rate (more than 65%) would be achieved. So, 88 physicians (15% of the total; 44 cardiologists, 22 GPs, and 22 internists) were invited to participate in the study. The sample of physicians was random and stratified by specialty mainly involved in the management of hypertension in Cyprus and the total population of hypertensives attending physicians within these districts. More specifically, based on data provided from the CSH and the CSC, physicians involved in the management of hypertension were divided in three groups based on their specialty: cardiologists, general practitioners (GPs), and internists. Then, a certain number of physicians, proportional to the size of hypertensive population treated by each specialty, were randomly selected from each one of the three specialty groups within each district. According to CSH and the CSC, although the proportion of cardiologists, internists, and GPs in Cyprus is 1 : 2 : 2, respectively, they are involved in hypertension management in a proportion of 2 : 1 : 1, respectively. Each participating physician recruited a maximum of 10 participants.

Written informed consent (personally signed and dated) was obtained from the patient at the initial visit, prior to proceeding with the study. Patients were eligible if they were hypertensive (firstly or previously diagnosed, treated or untreated) and aged ≥18 years, with no recent acute coronary syndrome or stroke (less than six months), recent or programmed revascularization, end-stage renal disease, or known lethal disease (e.g., malignancy) with expected survival less than one year. All participants were followed for a period of 6 months. During the follow-up period, at least two visits (one at the middle and another at the end of the 6 month period) were conducted. During the six-month clinical followup, the physicians were just collecting data about the management of each patient without any additional intervention or change in their usual clinical practice.

The study was approved by the Institutional Review Boards and the Greek Competent Authorities (EOF) and was carried out in accordance with the Declaration of Helsinki (1989) of the World Medical Association.

### 2.2. Data Collection

The case report form (CRF) was separated in two sections. In the first section, the characteristics of the participating physicians were collected along with questions aiming to assess physicians' awareness and self-reported implementation of guidelines as well as their perceptions about the guidelines. Then, the positive attitude of physicians towards guidelines was defined as disagreement with all of the following statements: (a) guidelines are difficult to apply, (b) guidelines are difficult to remember, (c) they are an industrial product, and (d) doctor knows best.

In the second section, the demographic and lifestyle characteristics of the enrolled patients and their family and personal medical history regarding CVD or cardiovascular risk factors were recorded. Moreover, anthropometric characteristics such as weight, height, and waist circumference (WC) as well as other clinical characteristics such as total cholesterol (TC), low density lipoprotein cholesterol (LDL), high density lipoprotein cholesterol (HDL), triglycerides (TG), systolic (SBP) and diastolic (DBP) blood pressure, and fasting blood glucose (FG) were measured. Body mass index (BMI) was calculated as the ratio of weight (Kg) with height squared (m^2^). Finally, for each examined cardiometabolic factor, the established by the physician targets and physicians' recommendations such as lifestyle changes (i.e., sodium restriction, smoking cessation, physical activity, healthy diet, etc.) or/and drug treatment were also described. These data were collected in each clinical visit, according to the treating physician's clinical practice.

### 2.3. Definitions of Hypertension, Obesity, Dyslipidemia, Diabetes Mellitus, and Metabolic Syndrome

Patients based on their baseline SBP/DBP levels were classified as follows [14]: optimal blood pressure: <120 and <80 mm Hg; normal: 120–129 and/or 80–84 mm Hg; high normal: 130–139 and/or 85–89 mm Hg; Grade I hypertension: 140–159 and/or DBP: 90–99 mm Hg; Grade II hypertension: 160–179 and/or DBP: 100–109 mm Hg; Grade III hypertension: >180 and/or DBP > 110 mm Hg. Obesity was defined as BMI ≥ 30 Kg/m^2^, while overweight was defined as those with 25 ≤ BMI < 30 Kg/m^2^. Patients were considered to have dyslipidemia when: (i) LDL-C levels were >3.0 mmol/L (115 mg/dL), (ii) HDL-C levels were <1.0 mmol/L (40 mg/dL) in men and <1.2 mmol/L (46 mg/dL) in women, (iii) TC > 5.0 mmol/L (190 mg/dL), (iv) TG > 1.7 mmol/L (150 mg/dL), or (iv) patients were on lipid-lowering treatment. As diabetics were considered, patients with FG greater than 125 mg/dL were on antidiabetic medication [14].

Finally, based on the new International Diabetes Federation (IDF) definition [22], patients were defined as having metabolic syndrome (MS) when they had central obesity (WC ≥ 94 cm in men and ≥80 cm in women) plus any two factors of the following four factors: (i) TG ≥ 1.7 mmol/L (150 mg/dL) or receiving lipid-lowering agents, (ii) HDL-C < 1.03 mmol/L (40 mg/dL) in men and <1.29 mmol/L (50 mg/dL) in women or receiving lipid-lowering agents, (iii) SBP ≥ 130 or DBP ≥ 85 mm Hg or receiving blood lowering medication, and (iv) FG ≥ 5.6 mmol/L (100 mg/dL) or previously diagnosed type 2 diabetes.

### 2.4. Adherence to Guidelines

Although there are plenty of published guidelines for the management of hypertension, the 2007 ESH/ESC guidelines were considered to be the most appropriate “gold standard” tool for the management of hypertension in the present study [14]. Based on these guidelines, patients were divided into two groups at the initial evaluation: those who ought to receive medication along with lifestyle recommendations for the management of hypertension and those who ought to adopt only some lifestyle recommendations. In particular, patients with Grade III hypertension should receive medication irrespective of the coexistence of other risk factors. Patients with Grade I and II hypertension should be treated with antihypertensive drugs if at least three additional risk factors or diabetes or MS or organ damage or established CVD or renal disease are presented, or if blood pressure remained uncontrolled after several weeks of lifestyle changes. Patients with high normal blood pressure should receive medication only if they were diabetics or if they had established CVD or renal disease. Patients with normal blood pressure should receive medication only if they had established CVD or renal disease according with the 2007 ESH/ESC guidelines. The rest of patients should get recommendations to adopt lifestyle changes. For previously diagnosed hypertensive patients with controlled blood pressure at baseline, it was considered that they ought to receive medication if they had diabetes mellitus, MS, established CVD, or renal disease or were smokers, dyslipidemic, and obese, since there is no available data regarding the initial grade of hypertension.

Moreover, based on the collected data regarding the physicians' recommendations, patients were classified into those whose physicians recommended medication use along with lifestyle changes and those who just got advices for lifestyle changes. Combining the data of these two classification methods, three indicators were calculated: (a) the “overall agreement rate” that reflects the proportion of patients whose physician's practice was in absolute agreement with the European guidelines; (b) the proportion of patients achieving medication guidelines in agreement with the European guidelines; (c) the proportion of patients achieving only lifestyle recommendations in agreement with the European guidelines.

### 2.5. Statistical Analysis

Normality of distribution was evaluated through the Shapiro-Wilk test. Normally distributed continuous variables are presented as mean ± standard deviation, and skewed variables are presented as median and interquartile range, while categorical variables are summarized as absolute and relative (%) frequencies. The measures reflecting the degree of adherence to guidelines are presented as percentages (95% confidence interval).

The overall agreement between physicians' clinical practice and guidelines was assessed by calculating the kappa measure of agreement. Associations between categorical variables were tested by the use of contingency tables and the calculation of chi-square tests without the correction of continuity. The association between continuous variables and categorical characteristics with two categories was evaluated through Student's *t*-test or Mann-Whitney when continuous variables were normally distributed or skewed, respectively. The association between continuous variables and categorical characteristics with three categories was evaluated through one way analysis of variance (ANOVA) or Kruskal-Wallis when continuous variables were normally distributed or skewed, respectively. However, due to multiple comparisons, the Bonferroni correction was used in order to account for the increase in Type I error.

Simple and multiple logistic regression analysis was also performed to determine the association of several patients' characteristics with probability of receiving correctly medication. Because of the cluster design of the current study (group of patients were enrolled in the study by the same physician), the physicians were considered in these analyses as a cluster variable.

A probability value of 5% considered as statistically significant. STATA software was used for all the statistical calculations (version 8, 2003, STATA Corp, College Station, TX, USA).

## 3. Results

### 3.1. Characteristics of Participating Physicians and Patients

In total, 68 physicians (44.1% from Nicosia, 29.4% from Limassol, 14.7% from Larnaca, and 10.3% from Paphos) accepted the invitation and participated in the study, and so the participation rate was 77.3% of the invited and 11.7% of the total Cypriot cardiologists, internists, and GPs. The participating physicians recruited 654 hypertensive patients in the current study. The characteristics of the participating physicians and patients are presented in the Tables 1 and 2, respectively. No significant difference was detected in the baseline characteristics of the physicians, with the exception that GPs were less likely to be men and had attended fewer international congresses/courses compared to cardiologists and internists (Table 1). However, the patients treated by cardiologists were younger but had higher systolic blood pressure than the patients followed by the other physicians (Table 2). Concerning the case mix by specialty (Table 3), although it seems that there was no difference in the number of treated patients with high cardiovascular risk (*P* > 0.22), internists have treated more than double patients with diabetes (*P* < 0.001) and chronic kidney disease (*P* = 0.02) and cardiologists have treated more than double patients with coronary artery disease (*P* < 0.001).

### 3.2. Self-Reported Awareness, Perception, and Implementation

Although 95.6% of the physicians reported that they were aware of some guidelines (Table 4), only 58.9% reported that they were specifically aware of the ESH/ESC guidelines and 25% of the JNC-7 report. The vast majority of participated physicians declared that they also use the respective guidelines (92.5%, Table 4). No statistically significant difference was detected in the self-reported awareness and implementation of guidelines among the three specialties of physicians (Table 4). Only 60.3% and 23.5% of the physicians reported that they were aware of two and three different scientific guidelines (ESH/ESC, BSH, JNC, WHO/ISH, etc.) for the management of hypertension, respectively. The results were consistent for all the specialties with the exception that it was easier for the cardiologists to apply these guidelines (*P* = 0.01) in comparison with internists and GPs. Finally, almost 72% of physicians declared that the guidelines are useful in practice and almost 63% of those reported that the guidelines are helpful in the effective management of hypertension (Table 4).

### 3.3. Adherence to Guidelines

Based on the European guidelines, 81.1% of patients ought to receive medication along with lifestyle recommendations and the rest of the patients to adopt only lifestyle changes. Cypriot physicians set the correct blood pressure targets in 95% of the patients and recommended lifestyle changes in almost all of them. Among lifestyle recommendations, the most usual were modification of their nutrition (91.6%), smoking cessation (90.9%), increase in physical activity (88.4%), salt restriction (83%), and weight loss (73.7%). Moreover, Cypriot physicians prescribed drugs at 66.6% of participated patients.

The overall adherence of physicians to the European guidelines (overall agreement rate) was 70.4% (*k* = 0.258, *P* < 0.001). Moreover, 72.9% of the patients were correctly advised by their physician to receive antihypertensive treatment to control their blood pressure, while 60.5% of the patients correctly got advices to adopt only lifestyle changes (Table 5). As concerns the effect of patients' characteristics on physicians' adherence to the European guidelines, it was found that the overall agreement rate ranges between 62.8% in patients with normal weight and 84.2% in patients with renal disease. The overall agreement rate was found to be significantly higher only in previously diagnosed patients with hypertension compared to the newly diagnosed ones and in obese patients compared to normal weight. The percent of patients correctly achieving medication guidelines was found to range between 61.9% and 84.2% in patients consuming ≥1 glass/day alcohol and in patients with renal disease, respectively. This percentage was significantly higher in previously diagnosed patients with hypertension compared to the newly diagnosed ones, and in those with low (no education or primary education) or moderate (secondary) educational status compared to those with high educational status (tertiary). Finally, the percent of patients achieving only the lifestyle guidelines in agreement with the European guidelines ranges between 44.7% and 83.3% in old cases and in patients with three risk factors (smokers, dyslipidemic, and obese), respectively. This percentage was found to be significantly higher among newly diagnosed patients and in younger patients (<55 years old for men and <65 years old for women) (Table 5).

Logistic regression analysis was conducted in order to determine the effect of patients' characteristics on the probability of correctly receiving antihypertensive medication after taking into account that patients have been selected as clusters of physicians. Among the investigated characteristics, only the diagnostic status of hypertension (i.e., old or new case) and patient's educational status were found to be independently associated with the correct use of medication for the management of hypertension. In particular, it was found that newly diagnosed patients were almost 60% less likely to correctly receive medication compared to previously diagnosed patients (OR: 0.40 (0.23–0.66), *P* = 0.001), while patients with high educational status were almost 50% less likely to correctly receive medication compared to those with low educational status (OR: 0.51 (0.29–0.90), *P* = 0.020). 

In order to evaluate the effect of physicians' characteristics on degree of their adherence to the European guidelines, the “level of agreement,” the “percent of patients correctly achieving medication guidelines,” and the “percent of patients correctly achieving only lifestyle guidelines” were calculated for each physician, separately. The percent of patients correctly achieving medication guidelines was found to be significantly higher among women, GPs, those attending more than 5 local conferences annually, and those working in public sectors compared to their counterparts (Table 6). However, among the different physicians' characteristics there was no statistically significant difference both in “the overall agreement rate” and the percent of patients achieving lifestyle guidelines (Table 6).

Moreover, adherence to guidelines was not related with better blood pressure control, with the exception of the cardiologists correctly recommending lifestyle modification who achieved better blood pressure control than those who did not (89.3% versus 61.9%, *P* = 0.023) (Table 7). The internists and the patients with high cardiovascular risk or obesity demonstrated a poor blood pressure control (Table 7). None of the other physicians' or patients' characteristics were related to blood pressure control.

## 4. Discussion

Clinical guidelines for the treatment of hypertension have been developed aiming to increase the number of hypertensive patients detected and effectively treated; however, their implementation in clinical practice is largely neglected by physicians [19]. The aim of the current study was to assess the degree of physicians' adherence to the European guidelines for the management of hypertension as well as the factors that may affect physicians' adherence in Cyprus. Physicians' adherence was determined by comparing the actual physicians' recommendations with the expected recommendations based on the guidelines. At this point, it should be noted that the European guidelines were used as the gold standard instead of JNC-7 and WHO/ISH guidelines because the former guidelines seem to be more relevant to the Cypriot population, are more updated than the last ones, and are supported by the relevant national scientific associations (CSH and CSC). Moreover, according to experts from these associations the Cypriot physicians are more familiar with these guidelines.

The results of the present study indicate that although the vast majority of physicians self-reported that they were aware of hypertension guidelines and they implemented these guidlines in daily practice, an overall agreement between physicians' practice and the European guidelines was detected in less than three-fourth of the hypertensive patients (70.4%). However, physicians' adherence to the guidelines was higher among hypertensive patients who ought to receive drug treatment based on the guidelines compared to the patients who ought to get only advices for lifestyle changes. In particular, it was found that the majority of the hypertensive patients (almost 73%) have been correctly advised to receive antihypertensive drug treatment, while the rest of the patients remained untreated resulting in an increased risk for CVD morbidity and mortality. Moreover, the percentage of correctly treated patients with antihypertensive treatment was found to be higher in patients with lower educational status, and in previously diagnosed hypertensive patients. On the other hand, it was found that almost 60% of patients got correctly advices to adopt lifestyle changes, while the rest of the actually low risk patients were treated with antihypertensive agents indicating a low degree of physicians' adherence to hypertension guidelines among low risk hypertensive patients. The high unreasonable use of antihypertensive agents observed in the present study among low-risk patients could be partially attributed firstly to physicians' perception that implementation of guideline recommendations will not lead to desired outcome and secondly to physicians perception that patients will not be adherent to their advices for lifestyle changes. The higher overall agreement rate and the percent of patients correctly achieving medication guidelines in previously diagnosed hypertensives is reasonable as there was more time to be detected, evaluated, and treated. On the contrary, the percent of patients correctly achieving only lifestyle guidelines was higher in newly diagnosed patients. Although the percent of patients correctly achieving medication guidelines was higher among GPs, probably due to time constraints and a low priority for hypertension at the patient's visit to the other specialties, the overall agreement rate was equal among the three specialties. The overall agreement rate was also equal among the other examined physicians' characteristics, but the percent of patients correctly achieving medication guidelines was higher among physicians working in public sector and attending more conferences, probably due to better continuing medical education. Finally, adherence to guidelines was not related with better blood pressure control in this study and more than half of the high risk patients were uncontrolled.

Several potential reasons for low physicians' adherence to hypertension guidelines have been reported in other relative studies, such as (1) physicians' unawareness of recommendations, (2) their disagreement with the guidelines, (3) the gap between the guideline committee experts and the physicians, (4) their perceptions that the guidelines are not easily applicable in daily practice, useful, and effective, (5) a suspicion in physicians' minds that a particular set of guidelines may have been excessively influenced by scientific biases, (6) the high number of practice guidelines and the confusion that may be generated by even small differences in the recommendations, (7) the perception that a recorded blood pressure was not representative of the patient's typical blood pressure, (8) inability to overcome clinical inertia of previous practice, or a lack of motivation to change, (9) lack of outcome expectancy, (10) perception of the physicians for patients' adherence to recommendations for lifestyle changes, (11) several patients' characteristics, and finally (12) external barriers, including a lack of resources, time constraints and lack of a patient reminder system [23–25].

Although the present results are in agreement with those of previous similar studies indicating the low degree of physicians' adherence to guidelines [26–29], our findings are not comparable with those of other studies since different methodologies have been used to assess physicians' adherence [19]. A review of similar studies in the international literature suggests that due to its multifactorial aspect, measuring doctors' adherence to guidelines is a concept (and an exercise) that can be translated methodologically in a number of different ways, depending largely on the study design (e.g., retrospective chart review of patient records, cross-sectional population-based studies, prospective “real life” management of patients such as the case of this study, etc.) as well as the range of available data and the level of detail introduced in the analysis [19]. Some population-based or record studies have simply quantified the extent to which doctors perform the necessary clinical examinations/laboratory tests [30]. This was out of the scope of the current study since our aim was to evaluate the degree of physicians' adherence to guidelines for management of hypertension and not for diagnosis of hypertension. Furthermore, collecting the necessary information and/or performing the right tests is not necessarily an indication of successful recognition of risk and even less so assignment of right treatment. Alternatively, studies have investigated the degree of agreement between the targets as set by doctors (for SBP, lipids, etc.) and recommended guideline targets [27]. A potential problem is that doctors may often set “realistic” targets that patients can achieve in the short period between follow-up visits rather than “ultimate” goals.

The results of the present study may be affected by several limitations. First of all the small sample size of physicians participated in the study may explain the lack of statistically significant associations between physicians' characteristics and the degree of their adherence to the European Guidelines. Secondly, in the present study, the physicians' recommendations were used as a sole measure of adherence. Ideally physicians' compliance should be examined by using a more composite indicator that would evaluate type of treatment, achievement of blood pressure goals, followup, and monitoring, respectively.

## 5. Conclusion

In summary, the findings of the present study indicate that although the vast majority of Cypriot physicians self-reported that they are aware of and implement hypertension guidelines in daily practice, a significantly lower agreement rate between physicians' practice and European guidelines was detected. In particular, it was found that more than one-fourth of high risk hypertensive patients remained untreated, half of them remained uncontrolled, and almost 40% of low risk patients received medications unreasonably. Bearing in mind that untreated and/or uncontrolled hypertension in high risk population is associated with increased cardiovascular morbidity and mortality and unreasonable use of antihypertensive treatment is associated with raised health expenditures, there is a strong need to draw and implement programs aiming to raise the awareness of physicians regarding the benefits of guidelines implementation.

## Figures and Tables

**Table 1:** Characteristics of the physicians.

	Total *N* = 68	Cardiologists *N* = 36	Internists *N* = 13	GPs *N* = 19	*P* value*
Age (years)^†^	46.5 (8.3)	46.8 (7.5)	42.9 (6.5)	46.5 (10.2)	0.393
Gender^§^					
Male	45 (66.2%)	28 (77.8%)	9 (69.2%)	8 (42.1%)	0.032
Hospital type^§^					
Public sector	33 (50%)	15 (41.7%)	7 (53.8%)	11 (64.7%)	0.281
Position of physician^§^					
Specialists	60 (93.8%)	34 (97.1%)	12 (92.3%)	14 (87.5%)	0.410
Years of experience among specialists^§^					
More than 10 years	31 (49.2%)	17 (51.5%)	4 (33.3%)	10 (55.6%)	0.465
Further education^§^					
MSc or PhD	16 (23.5%)	11 (31.4%)	3 (23.1%)	2 (10.5%)	0.235
Annual attendance of					
National medical congresses/courses^‡^	8 (3–11)	8 (3–15)	8 (6–13)	10 (3–10)	0.944
International medical congresses/courses^‡^	3 (2–6)	4 (2–9)	4 (3–9)	1 (0–4)	0.004

^†^Data are presented as mean (SD).

^‡^Data are presented as median, (Interquartile range).

^§^Data are presented as absolute and relative frequencies.

**Table 2:** Baseline characteristics of the patients.

	Total *N* = 654	Cardiologists *N* = 352	Internists *N* = 120	GPs *N* = 182	*P* value
Age (years)^†^	55.6 (12.7)	54.3 (13.5)	56.3 (11.1)	57.5 (11.9)*	0.018
SBP (mmHg)^†^	146.8 (23.4)	149.5 (23.0)	143.6 (22.9)	143.7 (24.0)*	0.006
DBP (mmHg)^†^	88.7 (12.2)	89.3 (11.9)	87.8 (12.2)	88.1 (12.9)	0.363
Body mass index (kg/m^2^)^†^	29.6 (6.2)	29.3 (5.9)	30.6 (6.6)	29.7 (6.3)	0.174
Waist circumference (cm)^†^	100.5 (16.9)	99.8 (14.1)	100.8 (23.3)	101.9 (16.6)	0.510
Total cholesterol^†^	231.7 (50.1)	232.1 (48.9)	233.5 (54.2)	229.7 (49.7)	0.806
LDL^†^	148.1 (43.6)	148.2 (44.1)	149.0 (46.6)	147.3 (40.7)	0.951
HDL^†^	50.8 (23.7)	52.8 (29.8)	47.7 (13.5)	49.0 (13.2)	0.071
Triglycerides^†^	166.1 (92.9)	165.0 (88.0)	169.0 (105.4)	166.4 (93.8)	0.927
Glucose^†^	109.2 (38.0)	107.8 (35.8)^¶^	119.5 (48.7)	105.2 (32.7)^¶^	0.004
Newly diagnosed (%)	338 (51.7%)	180 (51.1%)	63 (52.5%)	95 (52.2%)	0.954
Educational level (%)					
None/primary	199 (30.7%)	87 (25.1%)	41 (34.5%)	71 (39.0%)	
Secondary	252 (38.9%)	137 (39.5%)	43 (36.1%)	72 (39.6%)	0.003
Tertiary	197 (30.4%)	123 (35.4%)	35 (29.4%)	39 (21.4%)	
Smoking (%)					
Never	350 (54.4%)	198 (57.4%)	57 (47.9%)	95 (53.1%)	
Past	152 (23.6%)	68 (19.7%)	32 (26.9%)	52 (29.1%)	0.071
Current	141 (21.9%)	79 (22.9)	30 (25.2%)	32 (17.9%)	
Physical exercise (%)					
Never	267 (41.3%)	143 (41.2%)	56 (47.9%)	68 (37.4%)	
1-2 times per week	200 (31.0%)	105 (30.3%)	29 (24.8%)	66 (36.3%)	0.262
≥3 times per week	179 (27.7%)	99 (28.5%)	32 (27.4%)	48 (26.4%)	
Alcohol consumption (%)					
None	337 (52.7%)	172 (49.9%)	76 (64.4%)	89 (50.3%)	
<1 glass/day	199 (31.1%)	112 (32.5%)	31 (26.3%)	56 (31.6%)	0.057
>1 glass/day	104 (16.3%)	61 (17.7%)	11 (9.3%)	32 (18.1%)	

^†^Data are presented as mean (standard deviation).

*P < 0.05 for comparison with cardiologists taking into account the Bonferroni correction.

^¶^ *P* < 0.05 for comparison with GPs taking into account the Bonferroni correction.

**Table 3:** Case mix by specialty*.

	Total *N* = 654	Cardiologists *N* = 352	Internists *N* = 120	GPs *N* = 182	*P* value
High cardiovascular risk^†^	494 (75.5%)	260 (73.9%)	99 (82.5%)	135 (74.2%)	0.22
Diabetes mellitus	123 (18.8%)	56 (15.9%)	40 (33.3%)	27 (14.85)	<0.001
Chronic kidney disease	20 (3.1%)	6 (1.7%)	8 (6.7%)	6 (3.3%)	0.02
CVD	116 (17.7%)	75 (21.3%)	17 (14.2%)	24 (13.2%)	0.04
Coronary artery disease	96 (14.7%)	66 (18.8%)	11 (9.2%)	19 (10.4%)	<0.01
Stroke	21 (3.2%)	12 (3.4%)	5 (4.2%)	4 (2.2%)	0.61
Peripheral artery disease	14 (2.1%)	4 (1.1%)	1 (0.8%)	9 (5.0%)	0.01

*Data are presented as absolute and relative frequencies.

^†^High cardiovascular risk indicates the presence of any of the following: established cardiovascular disease, chronic kidney disease, diabetes mellitus, metabolic syndrome, or three or more risk factors [14].

**Table 4:** Physicians' awareness of and perception about the published guidelines for the management of arterial hypertension by specialty.

	Total *N* = 68	Cardiologists *N* = 36	Internists *N* = 13	GPs *N* = 19	*P* value
Self-reported awareness	65 (95.6%)	35 (97.2%)	13 (100.0%)	17 (89.5%)	0.28^†^
Self-reported adherence	62 (92.5%)	34 (94.4%)	13 (100.0%)	15 (83.3%)	0.18^†^
Physicians' opinion about guidelines					
Useful in practice	49 (72.1%)	27 (75.0%)	9 (69.2%)	13 (68.4%)	0.85
Helpful in effective management	43 (63.2%)	24 (66.7%)	7 (53.8%)	12 (63.2%)	0.71
Not useful	3 (4.4%)	3 (8.3%)	0 (0%)	0 (0%)	0.25
Difficult to apply	5 (7.4%)	0 (0.0%)	3 (23.1%)	2 (10.5%)	0.01^†^
Difficult to remember	3 (4.4%)	3 (8.3%)	0 (0.0%)	0 (0.0%)	0.42^†^
Industrial product	5 (7.4%)	1 (2.8%)	2 (15.4%)	2 (10.5%)	0.20^†^
Doctor knows always best	8 (11.8%)	3 (8.3%)	1 (7.7%)	4 (21.1%)	0.42^†^
Positive general attitude*	51 (75.0%)	30 (83.3%)	9 (69.2%)	12 (63.2%)	0.23

^†^Fisher's exact test, in all other cases, *χ* ^2^ was applied.

*A positive attitude was defined as disagreement with all of the following statements: (a) guidelines are difficult to apply, (b) guidelines are difficult to remember, (c) they are an industrial product, and (d) doctor knows best.

**Table 5:** Patients' characteristics and level of physicians' adherence to European guidelines.

	Level of agreement % (95% CI)	Medication guideline % (95% CI)	Lifestyle guideline % (95% CI)
Overall	70.4 (66.6–74.0)	72.9 (68.7–76.7)	60.5 (50.9–69.6)
Established CVD			
No	71.0 (66.8–74.9)	74.3 (66.8–74.9)	60.5 (50.9–69.6)
Yes	67.6 (57.8–76.4)	67.6 (57.8–76.4)	
Renal disease			
No	70.0 (66.0–73.7)	72.4 (68.1–76.4)	60.5 (50.9–69.6)
Yes	84.2 (60.4–96.6)	84.2 (60.4–96.6)	—
Diabetes			
No	69.6 (65.1–73.9)	72.6 (67.7–77.2)	59.4 (48.9–69.3)
Yes	72.6 (64.9–79.4)	73.3 (65.2–80.5)	66.7 (41.0–86.7)
MS			
No	67.3 (62.0–72.2)	71.2 (65.3–76.7)	55.6 (44.1–66.6)
Yes	74.3 (68.7–79.5)	74.6 (68.5–80.0)	72.7 (54.5–86.7)
Smokers and dyslipidemic and obese			
No	69.8 (65.8–73.6)	72.5 (68.1–76.6)	59.3 (49.4–68.6)
Yes	76.9 (63.2–87.5)	76.1 (61.2–87.4)	83.3 (35.9–99.6)
Gender			
Male	70.0 (65.0–74.7)	71.0 (65.4–76.2)	66.7 (54.3–77.6)
Female	70.8 (64.6–76.5)	75.5 (68.9–81.4)	50.0 (34.6–65.4)
Diagnosis status			
Old case	74.9 (69.4–79.9)*	81.4 (75.7–86.2)*	44.7 (30.2–59.9)*
New case	65.9 (60.3–71.2)	64.5 (58.1–70.5)	71.7 (58.6–82.5)
Smoking status			
Never smoker	69.3 (63.9–74.3)	72.4 (66.5–77.7)	57.8 (44.8–70.0)
Former smoker	72.3 (64.2–79.5)	76.5 (67.7–83.9)	53.8 (33.4–73.4)
Current smoker	70.5 (61.9–78.1)	69.7 (60.2–78.2)	73.9 (51.6–89.7)
BMI status			
Normal weight	62.8 (51.7–72.9)*	65.0 (51.6–76.9)	57.7 (36.9–76.6)
Overweight	68.8 (62.9–74.3)	71.4 (64.8–77.4)	60.0 (45.9–73.0)
Obese	74.5 (68.6–79.8)	63.6 (45.1–79.6)	63.6 (45.1–79.6)
Educational status			
None/primary	72.9 (65.9–79.1)	77.7 (70.1–84.1)*	56.4 (39.6–72.2)
Secondary	69.3 (63.0–75.1)	71.3 (64.5–77.4)	58.3 (40.8–74.5)
Tertiary	65.5 (57.2–73.2)	65.5 (56.0–74.2)	65.6 (46.8–81.4)
Physical activity status			
Never	70.9 (65.0–76.3)	74.7 (68.3–80.3)	53.5 (37.7–68.8)
1-2 times/week	70.4 (63.1–77.0)	72.3 (64.2–79.5)	63.2 (46.0–78.2)
≥3 times/week	69.6 (61.8–76.7)	70.4 (61.6–78.2)	66.7 (48.2–82.0)
Alcohol consumption			
Never	69.3 (63.8–74.3)	72.8 (66.7–78.2)	56.5 (43.3–69.0)
<1 glass/day	70.2 (63.1–76.6)	70.7 (62.9–77.7)	67.7 (48.6–83.3)
≥1 glass/day	74.1 (64.8–82.0)	61.9 (38.4–81.9)	61.9 (38.4–81.9)
Patients' age			
<55 (male) or 65 (female)	69.8 (64.7–74.4)	70.4 (64.8–75.6)	67.1 (55.1–77.7)*
>55 (male) or 65 (female)	70.5 (64.0–76.4)	75.4 (68.5–81.5)	47.2 (30.4–64.5)

MS: metabolic syndrome; BMI: body mass index; RF: risk factors; CVD: cardiovascular disease.

*P value < 0.05.

**Table 6:** Physicians' characteristics and level of physicians' adherence to European guidelines.

	Level of agreement (%) Mean (95% CI)	Medication guideline (%) Mean (95% CI)	Lifestyle guideline (%) Mean (95% CI)
Gender			
Male	69.0 (63.2–74.9)	68.6 (61.6–75.6)*	63.7 (48.2–79.2)
Female	73.5 (65.6–81.4)	80.8 (73.3–88.2)	53.4 (32.8–74.0)
Specialty			
Cardiologists	67.8 (61.5–74.1)	69.0 (61.5–76.4)*	52.4 (32.9–71.8)
GPs	78.4 (70.0–86.9)	83.8 (76.5–91.0)	67.3 (46.9–87.7)
Internists	66.6 (54.2–79.1)	67.1 (51.1–83.1)	63.3 (33.2–93.5)
Hospital type			
Public sector	74.3 (68.3–80.3)	79.2 (72.5–85.9)*	53.5 (35.2–71.9)
Private sector	67.4 (60.0–74.9)	66.4 (57.9–74.9)	67.4 (49.5–85.3)
Years of experience among specialists			
More than 10 years	69.5 (63.9–75.1)	74.2 (67.2–81.1)	61.9 (44.6–79.1)
Less than 10 years	72.4 (64.5–80.2)	72.2 (63.0–81.3)	58.7 (39.5–77.9)
Further education			
None	71.2 (65.8–76.7)	74.1 (67.9–80.3)	57.2 (43.1–71.3)
MSc or PhD	67.8 (57.8–77.7)	66.6 (55.6–77.6)	71.3 (45.1–97.4)
Physicians' opinion: guidelines are useful in practice			
No	65.7 (55.1–76.3)	65.0 (53.2–76.7)	63.1 (38.0–88.3)
Yes	72.4 (67.3–77.5)	75.8 (69.8–81.7)	57.9 (43.6–72.2)
Physicians' opinion: guidelines are helpful in effective management			
No	71.0 (63.3–78.7)	76.7 (69.3–84.2)	52.5 (33.5–71.4)
Yes	70.3 (64.3–76.3)	70.4 (63.0–77.8)	63.2 (47.1–79.3)
Physicians' opinion: positive general attitude			
No	72.4 (63.5–81.2)	68.7 (57.6–79.8)	73.1 (49.3–96.9)
Yes	69.9 (64.4–75.5)	74.0 (67.8–80.4)	54.6 (40.2–68.9)
ESH guidelines self-reported awareness			
No	67.8 (59.7–75.8)	73.5 (64.2–82.7)	51.3 (33.0–69.7)
Yes	72.6 (70.0–78.2)	72.2 (65.5–78.9)	65.1 (48.5–81.7)
Number of local conferences			
<5	66.2 (58.4–74.1)	66.0 (55.7–76.4)*	64.0 (45.3–82.6)
>5	73.2 (67.4–79.0)	76.9 (71.0–82.7)	56.4 (40.0–73.1)
Number of international conferences			
<5	69.6 (64.4–74.8)	72.0 (65.7–78.3)	58.7 (44.6–72.9)
>5	73.4 (62.4–84.3)	74.9 (63.9–85.9)	61.8 (35.0–88.6)

GP: general practitioner; *P < 0.05.

**Table 7:** Relation of adherence to guidelines, specialty, risk category, and BMI with blood pressure control.

	Uncontrolled	Controlled	*P* value
Adherence to guidelines			

Overall agreement			
No	66 (36.9%)	113 (63.1%)	0.617
Yes	148 (34.7%)	43 (65.3%)	
Medication guideline			
No	51 (38.3%)	82 (61.7%)	0.652
Yes	129 (36.1%)	228 (63.9%)	
Lifestyle guideline^†^			
No	14 (31.1%)	31 (68.9%)	0.681
Yes	19 (27.5%)	50 (72.5%)	

Other variables			

Specialty			
Cardiologists	106 (32.0%)	225 (68.0%)	
Internists	60 (53.6%)	52 (46.4%)	<0.001
GPs	48 (29.6%)	114 (70.4%)	
Cardiovascular risk			
High risk*	126 (53.4%)	110 (46.6%)	<0.001
Low-medium risk	88 (23.8%)	281 (76.2%)	
Body mass index (BMI)^§^			
Normal	22 (25.6%)	64 (74.4%)	
Overweight	85 (32.0%)	181 (68.0%)	0.008
Obese	105 (41.8%)	146 (58.2%)	

^†^Cardiologists who correctly recommended only lifestyle modification achieved better blood pressure control than those who did not (89.3% versus 61.9%, *P* = 0.023).

*High cardiovascular risk indicates the presence of any of the following: established cardiovascular disease, chronic kidney disease, diabetes mellitus, metabolic syndrome, or three or more risk factors [14].

^§^Normal was defined those with BMI < 25 Kg/m^2^, overweight those with 25 ≤ BMI < 29.9 Kg/m^2^, and obese those with BMI ≥ 30 Kg/m^2^.
